# Online Versus Telephone Methods to Recruit and Interview Older Gay and Bisexual Men Treated for Prostate Cancer: Findings from the Restore Study

**DOI:** 10.2196/cancer.5578

**Published:** 2016-07-19

**Authors:** BR Simon Rosser, Benjamin Capistrant

**Affiliations:** ^1^ University of Minnesota Division of Epidemiology and Community Health Minneapolis, MN United States

**Keywords:** Qualitative research, Aged, Gays

## Abstract

**Background:**

Recently, researchers have faced the challenge of conflicting recommendations for online versus traditional methods to recruit and interview older, sexual minority men. Older populations represent the cohort least likely to be online, necessitating the use of traditional research methods, such as telephone or in-person interviews. By contrast, gay and bisexual men represent a population of early adopters of new technology, both in general and for medical research. In a study of older gay and bisexual men with prostate cancer, we asked whether respondents preferred online versus offline methods for data collection. Given the paucity of research on how to recruit older gay and bisexual men in general, and older gay and bisexual men with prostate cancer in particular, we conducted an observational study to identify participant preferences when participating in research studies.

**Objective:**

To test online versus offline recruitment demographic data collection, and interview preferences of older gay and bisexual men with prostate cancer.

**Methods:**

Email blasts were sent from a website providing support services for gay and bisexual men with prostate cancer, supplemented with an email invitation from the web-host. All invitations provided information via the study website address and a toll-free telephone number. Study tasks included respondents being screened, giving informed consent, completing a short survey collecting demographic data, and a 60-75 minute telephone or Internet chat interview. All materials stressed that enrollees could participate in each task using either online methods or by telephone, whichever they preferred.

**Results:**

A total of 74 men were screened into the study, and 30 were interviewed. The average age of the participants was 63 years (standard deviation 6.9, range 48-75 years), with most residing in 14 American states, and one temporarily located overseas. For screening, consent, and the collection of demographic data, 97% (29/30) of the participants completed these tasks using online methods. For the interview, 97% (29/30) chose to be interviewed by telephone, rather than Internet chat.

**Conclusions:**

Older gay and bisexual men, when given choices, appear to prefer a mixed methods approach to qualitative investigations. For most aspects of the study, the older men chose online methods; the exception was the interview, in which case almost all preferred telephone. We speculate that a combination of the deeply personal nature of the topic (sexual effects of prostate cancer treatment), unfamiliarity with online chat, and possibly the subject burden involved in extensive typing contributed to the preference of telephone versus online chat. Recruitment of older men into this study showed good geographic diversity. We recommend that other qualitative researchers consider a mixed methods approach when recruiting older populations online.

## Introduction

The literature regarding the recruitment of older cohorts and sexual minorities reads as a study in contrasts. In 2014, while 86% of all adults reported being online, only 59% of seniors (age >65) were online [1]. Although 68% of Americans in their early 70s go online, Internet use drops to 47% after age 75 [1]. The implication for qualitative researchers is that traditional recruitment and data collection methods, such as telephone or in-person interviews, are preferred (or necessary) to recruit many older adults [2]. In addition, online research on older adults raises ethical considerations in at least four areas: (1) providing sufficient support to facilitate ongoing social interactions, (2) managing older adults’ expectations, (3) providing encouragement without coercion, and (4) responding to individual needs [3]. In contrast, the literature on gay, bisexual, and other men who have sex with men indicates that online methods are preferred and more effective for recruiting gay and bisexual men into studies [4,5]. Gay and bisexual men are a minority group identified as early adopters of new technology [6]. Given the popularity of apps and websites for dating and partner-seeking [4,7], and high rates of online pornography consumption [8], being online has become an integral part of the experience of being a gay or bisexual man in the United States [9] and other developed countries [10,11]. Multiple studies note a disproportionately high use of the Internet and apps by the youngest cohorts of gay and bisexual men [7,9]. This finding leads to the question of whether older cohorts of gay and bisexual men are better recruited using online or traditional methods.

Research on older gay and bisexual men is scarce, potentially contributing to undocumented health disparities [12]. Prostate cancer research is one such area, in which treatment outcomes appear worse for gay and bisexual men than other men [13-15], but there has been insufficient research to understand this phenomenon [16]. Detailed qualitative studies are needed to document the experience of gay and bisexual men with prostate cancer. Historically, recruiting gay and bisexual men with prostate cancer into studies has proven extremely challenging. Only three quantitative studies exist, each using small cohorts ranging from 89 to 111 participants [15,17,18]. Cancer registries do not routinely collect demographic data on sexual orientation, leaving this population relatively invisible. Similarly, in all but the largest cities, there are insufficient numbers of gay and bisexual men undergoing prostate cancer treatment to make tailored group support services feasible.

In designing a qualitative study of gay and bisexual men with prostate cancer, we encountered insufficient research to guide best practices. Given the lack of methodological studies of older gay and bisexual men, we conducted an observational study to identify and test the preferences of older gay and bisexual men.

## Methods

Study participants were recruited via *Malecare,* the largest men’s cancer support group (utilizing both online and in-person groups) and advocacy organization in the United States. Each year, an estimated 800 to 1000 gay and bisexual men with prostate cancer join *Malecare*. *Malecare* members received an email with information about this study, and the same information was included in *Malecare* ’s e-newsletter. Both invitations identified the *Restore* study, as funded the by National Cancer Institute at the National Institutes of Health, and its purpose as, “looking at how prostate cancer treatment affects gay and bisexual men, our life and sexual partners, and our family and friends who provide care for us during treatment.” Eligibility criteria included: adults aged >18; ability to speak English; identification as a gay, bisexual, or other man who has sex with men; diagnosed with, and treated for, prostate cancer; and resident of the United States. In addition, we stratified recruitment by major treatment type (surgery, radiation, or other) until saturation was reached. Given the high rates of radical prostatectomies, the stratification resulted in this group being capped at 19 participants.

In the email blast, potential participants were provided information that listed both the study website and a toll-free telephone number. Study tasks included respondents being screened, giving informed consent, completing a short survey collecting demographic data, and a 60-75 minute interview. All materials stressed that enrollees could participate in each task either by going online (to the website) or by telephone, *whichever they preferred*.

## Results

A total of 74 men were screened into the study, 30 completed the consent process, and all 30 were interviewed. Average age of the participants was 63 years (standard deviation 6.9), ranging from 48 to 75 years. One man was under 50, six were in their 50s, ten were aged 60-64, six were aged 66-69, and seven were in their 70s. Twenty-six participants described their race/ethnicity as white, three as African American, and one as Latino. Two of the men reported their Human Immunodeficiency Virus status as positive, one as unsure, and the remainder as negative. The participants resided in 13 states (Alabama, California, Florida, Georgia, Illinois, Kansas, Massachusetts, Minnesota, New York, Oklahoma, Rhode Island, Washington, and West Virginia); seven resided in New York, and one US resident was temporarily located in Europe. For screening, consent, and the collection of demographic data, 97% (29/30) of the participants completed these tasks using online methods. For the interview, 97% (29/30) chose to be interviewed by telephone, rather than online chat (with a different person absenting in each case).

## Discussion

Older gay and bisexual men, when given choices regarding participation in qualitative research, appear to prefer a mixed methods approach to qualitative investigations. For most aspects of the study, almost all gay and bisexual men chose online methods. This result is consistent with efficiency; when reading an email or newsletter, it is easier and faster to click on a link than to telephone a study. Consistent with best online practices [19], we designed the screener to lead into a description of the study, then several pages of consent, followed by a brief demographics survey (as one seamless unit). It is not surprising, therefore, that all but one gay or bisexual man completed this entire process online. Given our experience using online chat in other studies of gay and bisexual men [20], we expected more participants to choose this option. However, when given the option to be interviewed by telephone or online chat, all but one participant chose telephone. We speculate that a combination of the deeply personal nature of the topic (sexual effects of prostate cancer treatment), possible lack of familiarity with online chat, the anticipated subject burden involved in extensive typing in chat for 60-75 minutes, and/or slow Internet connection contributed to the participants choosing telephone over online chat. Given that multiple participants expressed appreciation for the opportunity to discuss their experience of having prostate cancer, and the sexual challenges that treatment entails, a desire to talk about this taboo topic may also have contributed to their decision.

We highlight the geographic diversity in the sample as a strength of online recruitment of older gay and bisexual men with prostate cancer. Similar to early studies of gay and bisexual men online [21,22], and studies of younger cohorts of gay and bisexual men [23], examination of the residential zip codes of participants demonstrated participation across all regions of the United States, and participation by rural as well as urban respondents.

This study had several limitations. First, this was a very small study focused on individual interviews, which we share to help other researchers proposing similar studies. Given the lack of studies on how to recruit older sexual minorities, we cannot know how generalizable these results are. Second, the older gay and bisexual men in this study were all recruited from a website. This detail likely biases findings towards online preferences, making the choice to be interviewed by telephone more apparent. Third, there are only a handful of websites offering support services to gay and bisexual men with prostate cancer *.* Choosing the largest of these websites made practical sense; however, we do not know how well members on this website reflect the broader population of gay and bisexual men with prostate cancer, or how well our findings might generalize to other websites or health conditions. Fourth *,* we did not ask participants why they chose their preferences, or the strength of their preference. We recommend that researchers consider adding both questions to advance research on methods. Finally, consistent with the Pew Internet and American Life Project’s results [1], none of our participants were older than 75 years. Researchers aiming to study gay and bisexual men older than 75 may need to use other methods to recruit and research this cohort.

### Conclusions

Although an unprecedented number of gay and bisexual men are reaching middle and old age, little is known about aging and age-related health conditions for sexual minority men. While new research efforts may emerge to address this evidence gap regarding healthy aging in this cohort, it remains unclear how best to identify, recruit, and include this population in social and biomedical research. This limitation is particularly true for Internet-based research efforts, which may be better at identifying and recruiting gay and bisexual men than the collection of qualitative interview data. Specifically, we recommend that qualitative researchers (and others interested in studying this cohort via online recruitment) consider a mixed methods approach to recruitment, but continue to use telephone or in-person methods to interview. To advance research methods, we encourage other researchers to set up naturalistic experiments to test research preferences, particularly for difficult to recruit populations.
